# Endoscopic endonasal treatment of Meckel’s cave epidermoid cysts: case series and systematic review

**DOI:** 10.1007/s10143-026-04180-6

**Published:** 2026-03-23

**Authors:** Alessandro Carretta, Marcello Magnani, Alessandra Eleuteri, Federica Guaraldi, Giacomo Sollini, Sofia Asioli, Arianna Rustici, Ernesto Pasquini, Matteo Zoli, Diego Mazzatenta

**Affiliations:** 1 Department of Biomedical and Neuromotor Sciences (DIBINEM), University of Bologna, Bologna, Italy; 2 Programma Neurochirurgia dell’Ipofisi – Pituitary Unit, IRCCS Istituto delle Scienze Neurologiche di Bologna, Ospedale Bellaria, Bologna, Italy; 3 ENT Unit, Azienda USL di Bologna, Ospedale Bellaria, Bologna, Italy; 4 Anatomic Pathology Unit, IRCCS Istituto delle Scienze Neurologiche di Bologna, Ospedale Bellaria, Bologna, Italy; 5 Neuroradiology Unit, IRCCS Istituto delle Scienze Neurologiche di Bologna, Ospedale Maggiore, Bologna, Italy

**Keywords:** Meckel’s cave, Epidermoid cyst, Endoscopic endonasal approach (EEA), Systematic review, Skull base

## Abstract

Meckel’s cave (MC) epidermoid cysts (EC) are rare lesions. Extended endoscopic endonasal approaches (EEA) to the ventral skull base recently opened a minimally invasive corridor to MC. The aim of this study was to report our surgical experience and review the concerning literature. Our institutional registry was retrospectively reviewed, and patients who underwent an endoscopic endonasal approach for MC EC from 1998 to 2025 were included. Cases involving alternative surgical approaches or lesions with different histological diagnoses were excluded. A PRISMA systematic review of the literature was performed. The cohort consisted of 4 patients who underwent 6 endoscopic endonasal procedures (one patient experienced two recurrences and underwent two additional EEAs). Complete resection of the cyst content was achieved in 4 procedures (66.7%). Mean surgical time was 111.3 ± 57 min. Mean post-operative hospital length of stay (LOS) was 5.2 ± 1.9 days. Main complications consisted in 2 cases of temporary post-operative worsening of V2 hypoesthesia and 1 CSF leak. The systematic review retrieved 17 papers reporting 17 patients who underwent surgical treatment of MC EC. The reported approach exploited a transcranial route in 12 cases (70.6%) and an endoscopic endonasal route in 5 cases (29.4%). ECs primarily arising from MC are rare lesions with only few cases reported in literature. EEA provides a valid corridor to perform an effective and safe resection, while its reduced invasiveness allows a fast recovery with a shorter LOS. Further studies, with larger caseloads, are warranted to confirm these results.

**Supplementary Information:**

The online version contains supplementary material available at 10.1007/s10143-026-04180-6.

## Introduction

 Meckel’s cave (MC) epidermoid cysts (EC) are rare congenital benign lesions, with a small number of case reports published in literature since their first account in 1971 [1, 2]. The cornerstone of their treatment is still their resection through a transcranial route, with approaches, mainly consisting of intradural or extradural subtemporal approaches [1–14]. The need for the complete resection of the cyst capsule has been debated in literature, with authors advocating for its complete resection to minimize the risk of recurrence [15–17]. Nevertheless, the capsule is often strictly adherent to critical neurovascular structure, as the Gasserian ganglion, and the risk to incur in new postoperative deficits can be significant [17]. In those cases, a small capsule remnant can be left to avoid permanent trigeminal hypoesthesia, potentially with neuralgia, with a subsequent strategy of strict neuroradiological follow–up [18–20]. In the recent years, since the seminal publication of Kassam et al. in 2009, the development and the validation of extended endoscopic endonasal (EEA) approaches to the ventral skull base opened an innovative, minimally invasive, route to MC [21–28]. This approach was proven to be feasible and effective in the treatment of MC tumors, but, since their rarity, the adoption of EEA for MC ECs has been scarcely reported. As a consequence, there is no consensus about the first – choice approach in the surgical treatment of those rare lesions [28–31]. 

The objective of the present study is to assess the outcome of the EEA for MC EC in our single Institution series, comparing our results with those reported in literature, collected by the means of a systematic review.

## Materials and methods

### Case series

**Fig. 1:**
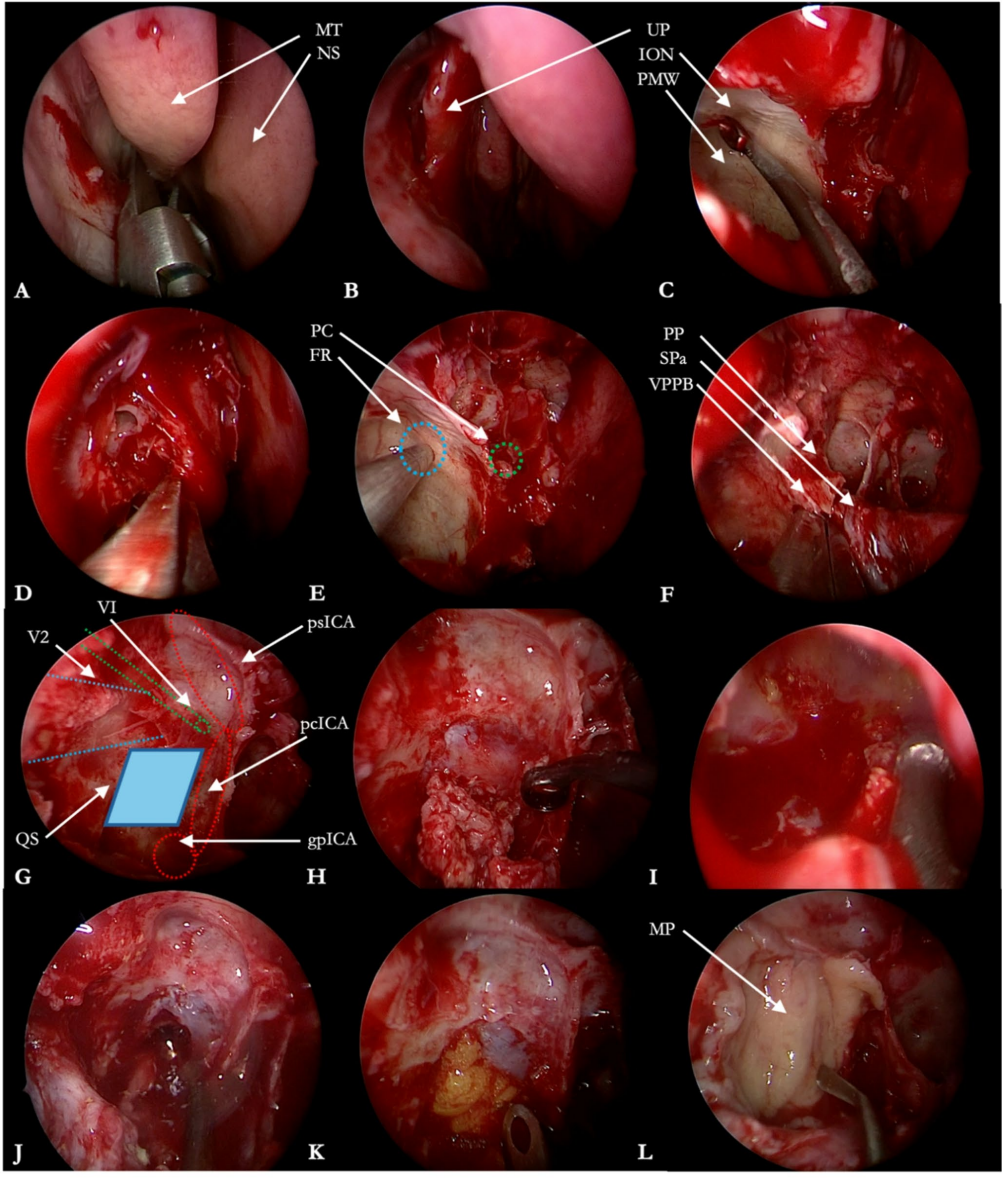
Staged removal of a Meckel’s cave epidermoid cyst. **A**. Middle turbinate resection. **B**. Inferior uncinectomy. **C**. Type A maxillectomy for the identification of the posterior maxillary sinus wall and infraorbital nerve impression on the roof of the sinus. **D**. Posterior ethmoidectomy. **E**. Neuronavigation-assisted identification of foramen rotundum and pterygopalatine canal. **F**. Dissection of the sphenopalatine canal and its content (i.e., sphenopalatine artery) as a landmark for the identification of the pterygopalatine canal, which lies posteriorly to it. **G**. Anatomical landmarks of the quadrangular space. Medially, paraclival ICA. Laterally, maxillary branch of trigeminal nerve impression on the lateral wall of the sphenoid sinus. Inferiorly, the genu between the petrous tract (C2) of the ICA and the paraclival tract (C3) of the ICA. Superiorly, abducens nerve (VI). **H**. After periosteal dura incision, epidermoid cyst is removed with the use of curettes. **I**. Surgical cave inspection with use of 30° endoscope and further epidermoid cyst removal with angled suction. **J**. Extensive irrigation of the surgical field to clean it from small remnants. **K**, **L**. Skull-base reconstruction with abdominal fat and mucoperiosteum free flap. gpICA, genu of petrous internal carotid artery. pcICA, paraclival internal carotid artery. psICA, parasellar internal carotid artery. FR, foramen rotundum. ION, infraorbital nerve. MP, mucoperiosteum. MT, middle turbinate. NS, nasal septum. PC, pterygopalatine canal. PMW, posterior maxillary wall. PP, pterygoid process. QS, quadrangular space. SPa, sphenopalatine artery. VPPB, vertical plate of palatine bone. UP, uncinate process. V2, maxillary branch of trigeminal nerve. VI, abducens nerve

**Fig. 2:**
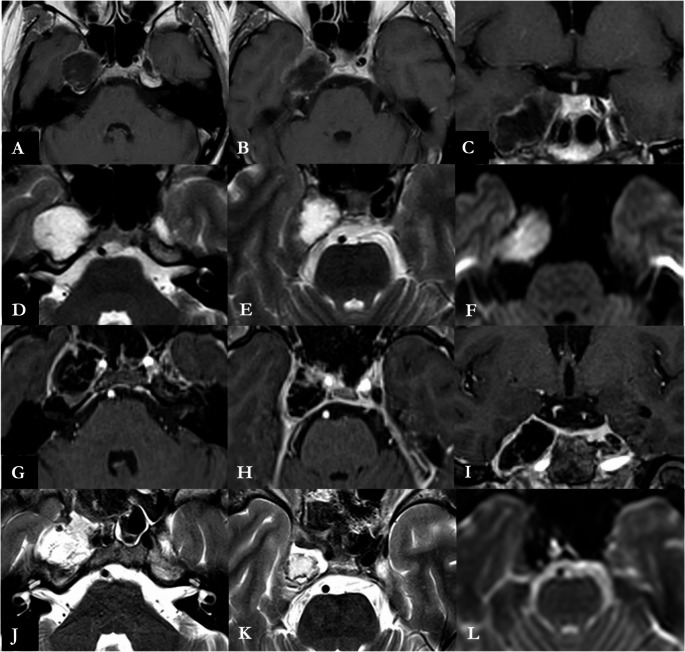
Preoperative axial (**A**, **B**) and coronal (**C**) contrast enhanced T1 – weighted, axial (**D**, **E**) T2 – weighted and axial (**F**) DWI MRI images showing a non – enhancing diffusion – restricting lesion of right Meckel’s cave. The postoperative axial (**G**, **H**) and coronal (**I**) contrast enhanced T1 – weighted, axial (**J**, **K**) T2 – weighted and axial (**L**) DWI MRI images reveal a gross – total resection of cystic componentPreoperative axial (**A**, **B**) and coronal (**C**) contrast enhanced T1 – weighted, axial (**D**, **E**) T2 – weighted and axial (**F**) DWI MRI images showing a non – enhancing diffusion – restricting lesion of right Meckel’s cave. The postoperative axial (**G**, **H**) and coronal (**I**) contrast enhanced T1 – weighted, axial (**J**, **K**) T2 – weighted and axial (**L**) DWI MRI images reveal a gross – total resection of cystic component

**Fig. 3:**
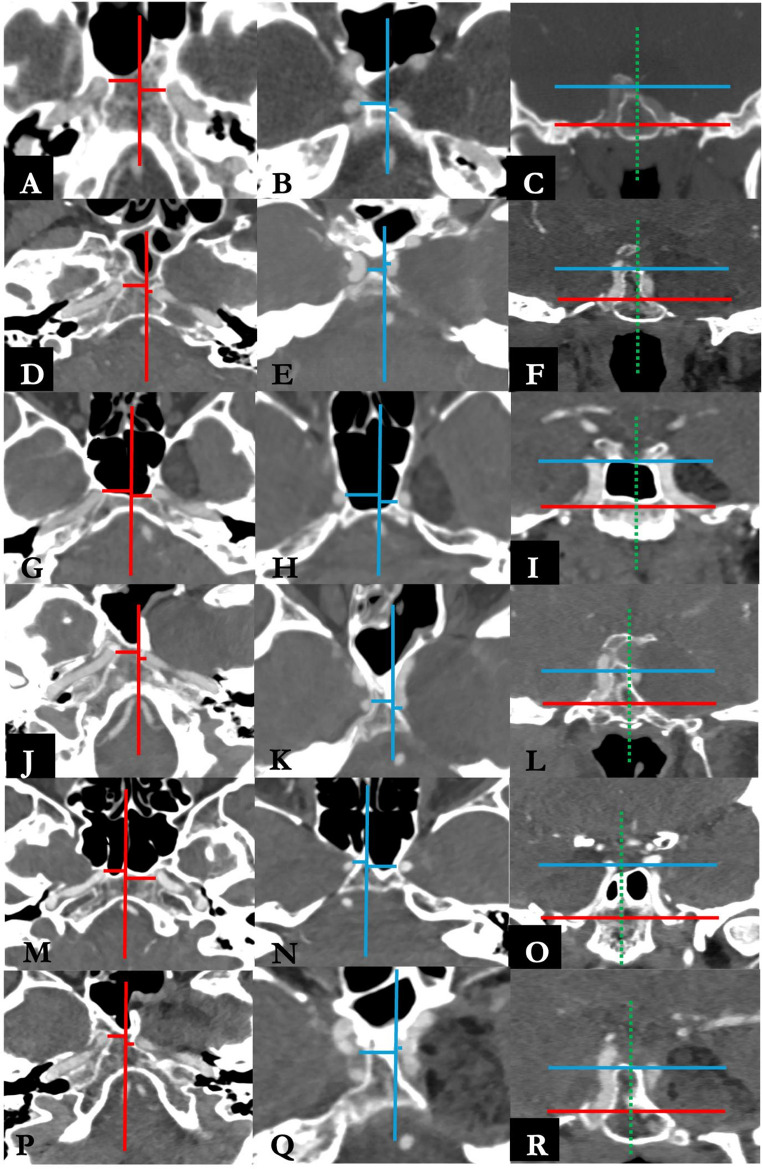
Preoperative angio-CT evaluation of ICA medialization in all the included procedures according to Zoli et al. [28] **A**, **B**, **C**: procedure #1. **D**, **E**, **F**: procedure #2. **G**, **H**, **I**: procedure # 3. **J**, **K**, **L**: procedure #4. **M**, **N**, **O**: procedure #5. **P**, **Q**, **R**: procedure #6. On the axial plane, two slices were selected at the level of C2-C3 genu (**A**, **D**, **G**, **J**, **M**, **P**) and C3-C4 genu (**B**, **E**, **H**, **K**, **N**, **Q**). On the coronal plane (**C**, **F**, **I**, **L**, **O**, **R**), the medialization of the ICA due to the presence of the lesion creates the surgical corridor to access Meckel’s cave.

The Institutional database of the Programma Neurochirurgia Ipofisi - Pituitary Unit of IRCCS Istituto delle Scienze Neurologiche di Bologna was retrospectively reviewed to include all the patients who underwent endoscopic endonasal treatment for MC EC between May 1998 (year of the first endoscopic skull base procedure in our center) and February 2025. Inclusion criteria consisted of histological confirmation of EC arising from MC, availability of medical reports, radiological imaging and a minimum follow–up of 3 months. Patients operated with other surgical approaches or with lesions with other histological diagnosis were excluded. According to our protocol, all patients underwent preoperative neurological examination, contrast – enhanced 1.5 or 3T MRI and CT angiogram. Surgical tenets and technique have been already published in detail elsewhere and an illustrative case is presented in Figs. 1 and 2 and Online Resource 1 [28]. Ipsilateral ICA medialization, a key of the endoscopic endonasal access to the MC, was qualitatively assessed on CT angiography according to Zoli et al. The ICA-to-midline distance was measured on standardized axial MPR slices at the C2–C3 and C3–C4 genu levels, and medial displacement when compared to contralateral ICA was observed, as reported in Fig. 3 [28]. Our main surgical goal was to drain the EC content. In those cases where the capsule appeared strictly adherent to the trigeminal nerves or the Gasserian ganglion and despite delicate bimanual dissection technique any traction was observed on neural structures, its removal was halted to avoid any direct injury, limiting to collect samples for histological purposes. Any coagulation of the capsule was likewise avoided, to prevent any thermal damage to neurovascular structures in a narrow surgical cave. After discharge, patients underwent an outpatient ENT assessment after one month, followed by 3 – months MRI, ophthalmological and neurosurgical evaluation. Further follow–up MRIs and neurosurgical evaluations were performed on a yearly basis.

Basing on medical records, pre-operative clinical and neuroradiological features, such as the volume of the cyst, as well as surgical time, complications and post-operative hospital length-of-stay (LOS) were recorded. Surgical outcome was evaluated at 3 months MRI to assess the extent of resection. Recurrences/progression were determined considering the following neuroradiological imaging, with a minimum follow – up of 3 months, defined as the *de novo* observation or volumetric increase of DWI – restricting tissue in the surgical cave when compared to the postoperative or previous follow – up exam. Gross total resection (GTR) was obtained if no remnant of cystic content was visible in T2 – weighted and DWI images at 3 - months MRI regardless of capsule resection [32], otherwise it was considered subtotal resection (STR) if the remnant was < 20% of initial volume or partial resection (PR) if the remnant was > 20% of initial volume. The volume of the lesion was estimated with the ABC/2 method (which, despite harboring unavoidable inaccuracy for lobulated neoplasms, can adequately approximate the volume of rounded lesions, such as ECs) on T2-weighted images and extent of resection was assessed on T2/DWI at 3 months.

For the patient who underwent multiple procedures, demographic data were counted once and clinical and surgical features were analyzed per single procedure.

Institutional review board and ethics committee approval was waived due to the retrospective observational nature of the study.

## Systematic review

A systematic review of literature was performed in accordance with the Preferred Reporting Items for Systematic Reviews and Meta-Analyses (PRISMA) statement guidelines. MEDLINE and SCOPUS databases were queried using individual keywords. The strings used for search were (“epidermoid” AND “Meckel” AND “cyst”), (“epidermoid” AND “Gasserian ganglion”), (“cholesteatoma” AND “Meckel”), (“cholesteatoma” AND “Gasserian Ganglion”). This search strategy was intentionally selected to prioritize specificity for epidermoid cysts primarily arising from Meckel’s cave. The results were then limited to the English language and human subjects. After duplicate removal, the titles and abstracts were first screened and, for the papers deemed appropriate, full texts were obtained and reviewed for appropriateness and the extraction of data. The articles’ reference lists were examined to identify any other relevant studies, including older articles using historical terminology (as “cholesteatoma” in the context of intracranial epidermoid lesions) [2]. The individual steps of title and abstract screening, full-text review, and data extraction were performed independently by two reviewers (A.C. and M.M.); disagreements at any stage were resolved by discussion and consensus, and the senior authors reviewed and approved the selection. The last search was performed on 24th December 2025.

The inclusion criterion was the report of any MC EC which underwent any type of surgical procedure, with any approach. The exclusion criteria were as follows: secondary MC involvement of large tumors primarily arising from other skull base regions (such as cerebello – pontine angle), and lack of data concerning type of preoperative symptomatology, surgical approach and complications.

Data from the included studies were extracted, organized, and analyzed in an ad – hoc database (Microsoft Excel 2019, Microsoft Corp, Redmond, WA, USA). The collected variables included the first author, publication year, country, age, sex, side, size, patients’ symptoms, type of approach, extent of resection, complications, symptoms outcome, lesion recurrence/progression and clinical status at follow-up.

## Results

### Case series

Four patients were included and outlined in Table 1 (one case already recurrent was operated twice with EEA for lesion progression after 4 and 6 years). No patients lacking pre - and postoperative data were excluded.

**Table 1:** clinical, radiological and surgical features of the included cohort, per single procedure

ID	Sex	Age	Side	Year of surgery	Symptoms	Volume cm^3^	Previous Surgery	Lenghth of surgery (minutes)	Reconstruction	EOR	Complications	LOS (days)	New postoperative deficits	Follow - up (months)	Deficit at F/U	EOR at F/U
1	M	26	R	2010	III CN Palsy	5.5	No	210	Fat, mucoperiostium	GTR	No	5	VI CN palsy	183	Regression of III CN palsy, persistence of VI CN palsy	GTR
2*	M	57	L	2019	Headache, diplopia, V1, V2, V3 hypoesthesia	15.8	Craniotomy 1 year. Earlier	146	Fat, nasoseptal flap	STR	No	4	III CN palsy	64	Regression of deficits and symptoms. Stable hypoesthesia.	Recurrence, operated
3	M	33	L	2023	V2 paresthesia	4.6	No	85	Fat, mucoperiostium	GTR	No	4	VI CN palsy	24	Regression of deficits and symptoms	GTR
4*	M	61	L	2023	Visual deficit, diplopia, trigeminal neuralgia, V1, V2, V3 hypoesthesia.	10.9	Craniotomy 5 yrs earlier, EEA 4 yrs earlier	82	Fat, nasoseptal flap	STR	CSF Leak	9	Worsening of V2 hypoesthesia.	19	Regression of deficits and symptoms. Stable hypoesthesia.	Recurrence, operated
5	F	46	R	2024	V2 hypoesthesia, dimensional increase of previous incidental finding	5.5	No	98	Fat, mucoperiostium	GTR	No	4	Worsening of V2 hypoesthesia.	12	Regression of deficits and symptoms	GTR
6*	M	62	L	2025	Trigeminal neuralgia, V1, V2, V3 hypoesthesia.	5.6	Craniotomy 7 yrs earlier, EEA 5 and 2 yrs earlier	47	Fat, nasoseptal flap	GTR	No	5	No	3	Regression of symptoms. Stable hypoesthesia.	GTR

Three patients were males and mean age at first surgery was 40.5 ± 13.8 years. One patient was previously operated via a fronto – orbito – zygomatic craniotomy in another Institution. Half of the ECs were in the left MC.

Mean preoperative lesion volume was 8 ± 4.5 cm^3^. Before 5 procedures (83.3%) patients complained of preoperative trigeminal hypoesthesia or paresthesia and in 2 (33.3%) of trigeminal neuralgia, while subjective diplopia or overt ophthalmoplegia was observed in 2 procedures (33.3%). Mean surgical time was 111.3 ± 57 min. In 3 patients osteodural defect reconstruction was performed using abdominal fat covered by a middle turbinate mucoperiosteum graft and in one using a nasoseptal flap, which was preserved and subsequently reused during the second and third surgical procedures for lesion progression respectively 4 and 6 years later. GTR was achieved in 4 (66.7%) procedures, and STR in 2 (33.3%), leaving a thin, millimetric, layer of neoplastic material strictly adherent to the capsule. Complete capsule removal was not achieved, instead a biopsy was performed in all instances. A postoperative CSF leak, which required an endoscopic endonasal revision procedure, was observed. Mean post-operative hospital length of stay was 5.2 ± 1.9 days. Neurological complications consisted in 2 transitory cranial nerve (CN) palsies, which fully recovered at follow - up and 1 permanent CN palsy. Temporary postoperative worsening of hypoesthesia in V2 territory was observed in 2 procedures (33.3%) and no *de novo* cases of trigeminal neuralgia were reported. No perioperative mortality occurred.

Mean follow – up time was 50.1 ± 68 months. At last follow – up, remission of preoperative trigeminal symptoms occurred in all patients. No recurrence was observed after a GTR. One lesion progression after STR was observed after 46 months and underwent a further endoscopic endonasal resection procedure (case #4), further achieving STR. The same patient experienced further dimensional increase of the residual lesion 16 months later and underwent another endoscopic endonasal resection procedure (case #6) ultimately achieving GTR.

## Illustrative case

A 46 years old female patient (case #5, Table 1), with no relevant comorbidities, was referred to our center harboring a 5.5 cm^3^ lesion in the right MC, with DWI – restricting signal consistent with a EC (Fig. 2). The lesion was incidentally diagnosed three years earlier, during a CT scan performed for mild traumatic brain injury. A wait-and-see approach was chosen, owing also to patient’s preferences, and she underwent yearly MRI follow – up imaging. The last MRI showed a slight but noticeable volumetric increase. The neurological examination was unremarkable, except for a mild right V_2_ hypoesthesia, developed six months earlier. The patient underwent an endoscopic endonasal approach (Fig. 1 and Online Resource 1) to resect the cyst. During the resection, strict adherences between the capsule and neural structures was observed and its removal was halted. Histopathological examination confirmed the diagnosis of epidermoid cyst and post – operative MRI acknowledged a GTR of the cystic component (Fig. 2). The patients complained a postoperative worsening of the right V_2_ hypoesthesia and was discharged at home on fourth postoperative day after an otherwise unremarkable clinical course. At 12 – months follow – up, the MRI confirmed a GTR with no recurrences and the V_2_ hypoesthesia, which was improving at 3 – months follow – up, completely regressed.

### Systematic review

**Fig. 4:**
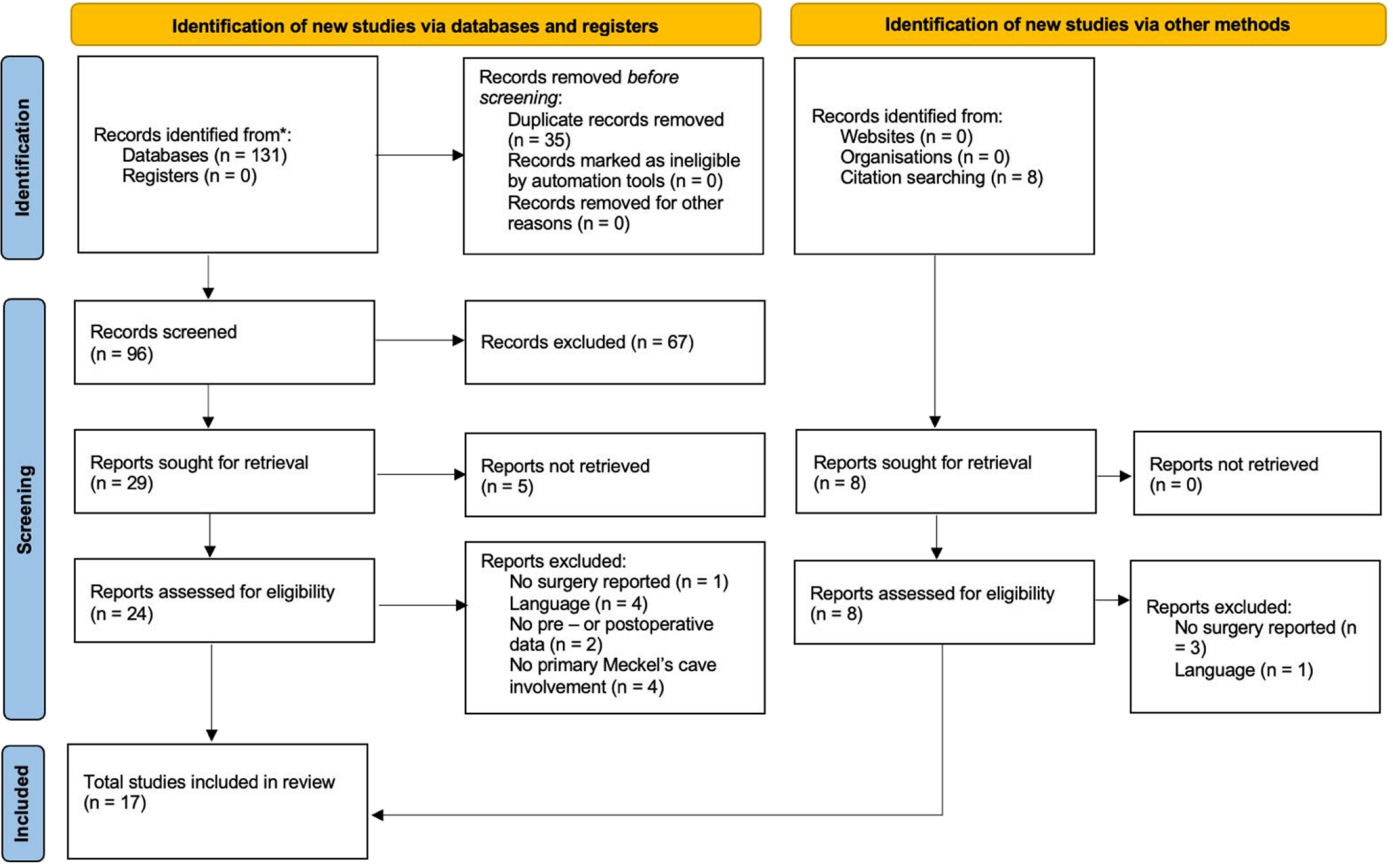
PRISMA flowchart of the systematic review

The systematic literature review retrieved 17 papers in a timespan from 1971 to 2024, including 17 patients operated for an EC primary arising from MC, and was outlined in Table 2; Fig. 4.

**Table 2:** Summary of included studies, describing preoperative clinical and radiological features, type of approach, extent of resection, complications, clinical outcome and follow–up

ID	Author	Year	Country	Age, sex	Side	Size	Symptoms	Surgical Approach	EOR	Complete capsule resection	Complications	Postoperative Outcome	Follow – up and clinical status
1	Mehta DS et al. [2]	1971	India	26, M	R	N/A	Trigeminal neuralgia	Intradural subtemporal	GTR	N/A	No	Improvement of trigeminal neuralgia, no new deficits.	N/A
2	Kapila A et al. [15]	1984	USA	62, F	R	N/A	Trigeminal neuralgia	Subtemporal	GTR	N/A	No	Improvement of trigeminal neuralgia, no new deficits.	N/A
3	Beck DW and Menenzes AH [4]	1987	USA	61, F	L	N/A	V, VII, VIII CN palsy	Intradural subtemporal	STR	No	No	Improvement of facial nerve deficit. No new deficits.	24 months, V and VIII CN palsy
4	Nadkarni T et al. [10]	2000	India	27, F	R	N/A	V CN palsy	“Interdural” subtemporal	GTR	N/A	No	Improvement of trigeminal neuralgia, no new deficits.	N/A
5	Furtado VS et al. [23]	2009	India	25, F	L	N/A	Trigeminal neuralgia	Extradural subtemporal	GTR	Yes	No	Improvement of trigeminal neuralgia, no new deficits.	12 months, V CN palsy
6	Arai et al. [3]	2010	Japan	27, F	R	N/A	V CN palsy	Extradural orbito - zygomatic	GTR	N/A	No	Worsening of V CN palsy	2 years, symptomatic improvement
7	Sun DQ et al. [12]	2017	USA	18, F	R	43 mm	Headache	Extradural extended middle cranial fossa, followed by EEA, followed by another extended middle cranial fossa	GTR	N/A	Seizures, CSF leak.	No improvement, mild cognitive difficulties, hearing loss.	105 months, stable
8	Zoli M et al. [28]	2018	Italy	26, M	R	5.5 cm3	V CN palsy	Endoscopic endonasal	GTR	No	No	Improvement of V CN palsy. New onset VI CN deficit.	24 months, regression of V CN palsy
9	Busch CM et al. [1]	2019	USA	51, F	L	1.9 cm3	III, V, VI CN palsy. Trigeminal neuralgia.	Extradural subtemporal	GTR	Yes	No	Improvement of trigeminal neuralgia, no new deficits.	12 months, V CN palsy
10	Gohil J et al. [5]	2019	India	43, M	L	N/A	V CN motor palsy	Intradural transsylvian	STR	No	No	N/A symptoms. No new deficits.	N/A
11	Zakaria J et al. [31]	2020	USA	64, F	R	21 × 13 mm	V CN palsy, dimensional increase of previous finding	Endoscopic endonasal	GTR	No	No	Trigeminal neuralgia	12 months, regression of neuralgia and V CN palsy
12	Aldea S et al. [29]	2021	France	34, F	R	N/A	V, VI CN palsy	Endoscopic endonasal	STR	No	No	Increase of trigeminal dysesthesias	N/A
13	Lopez Gomez P et al. [22]	2021	Spain	24, F	R	N/A	V CN palsy, gait imbalance	Intradural transzygomatic - subtemporal	GTR	N/A	No	Improvement of gait imbalance, no new deficits.	36 months, V CN palsy
14	Sharifi G et al. [11]	2022	Iran	32, M	L	30 mm	VI CN palsy	Extradural subtemporal	GTR	Yes	No	Improvement of abducens nerve deficit. No new deficits.	12 months
15	Morshed RA et al. [30]	2022	USA	43, F	L	N/A	V CN palsy. Trigeminal neuralgia.	Endoscopic endonasal	GTR	N/A	No	Stable	N/A
16	Morisako H et al. [9]	2024	Japan	48, M	R	40 mm	V CN palsy	Intradural endoscopic keyhole subtemporal	GTR	N/A	No	N/A symptoms. No new deficits	N/A
17	Ferlendis L et al. [33]	2024	Italy	47, F	L	20 × 14 mm	Trigeminal neuralgia, diplopia	Endoscopic endonasal	STR	N/A	No	Improvement of trigeminal neuralgia and diplopia, no new deficits.	N/A

Of those patients, 5 were males (29.4%) and the mean age was 38.7 ± 14.8 years. EC were located in the left side in 41.2% of cases. All cases were naïve for previous surgical or radiation therapies. Trigeminal hypoesthesia was reported in 10 cases (58.8%) and neuralgia in 6 cases (35.3%). Moreover, ophthalmoplegia was observed in 3 (17.6%) cases, a pure V CN motor palsy, and headache in 1 case (5.9%).

The first - choice surgical approach was a craniotomy, with different technical variations, in 12 patients (70.6%) and endoscopic endonasal in 5 patients (29.4%), all of them reported after 2018. Of note, a patient (#7) was operated three times, twice via an extended middle fossa approach and once via an endoscopic endonasal route. GTR was achieved in 13 cases (76.5%) and particularly in 60% of cases after an EEA. Cyst capsule degree of resection was reported in 8 cases, and complete resection was achieved in 3. Complications were observed in 1 case (#7, 5.9%), which experienced seizures and a CSF leak respectively after multiple craniotomies and an endoscopic endonasal procedure. Preoperative symptoms improved in 11 (64.7%) cases and in 3 (60%) after an EEA. Transitory or permanent CN deficits were observed in 4 cases (23.5%) and in 1 case (20%) after an EEA.

Follow – up data were available in 9 cases. Median follow – up time was 24 months (range 12–105). No reoperations (except for the case of patient #7) or recurrences were reported.

## Discussion

Our study analyzes a large single-center series of patients with MC ECs operated through an EEA, and this is, to our knowledge, the largest in literature. We observed the surgical advantages of this approach, given by its ventral route, which allows to approach the MC through a so – called “quadrangular space” (bounded medially by the paraclival C3 – C4 ICA, inferiorly by the petrous C2 ICA, laterally by V_2_ and superiorly by the VI CN, covering the anteromedial aspect of the Gasserian ganglion), can be helpful also in management of ECs [25, 28].

EEA achieved a satisfactory resection rate, obtaining a GTR of 66.7%. In all of the reported cases, the cyst capsule resection was precluded, due to strong adherences to the Gasserian ganglion and trigeminal roots, in order to avoid or accentuate postoperative deficits, as reported in literature [4, 17, 19, 34]. While incomplete removal of the cyst capsule has been suggested as a risk factor for recurrence [17, 34–37], , in our cohort recurrence occurred exclusively in two patients who had previously undergone transcranial surgery with subtotal resection. During procedures #2 and #4, from the ventral point of view granted by the endoscope, we observed tenacious scarring tissue and adherences of the epidermoid tissue and capsule in the lateral and dorsal aspects of the enlarged MC, where the previous transcranial approach entered the surgical cave, precluding therefore complete resection. As underlined by Vaz – Guimaraes et al., transcranial microsurgical approaches may give the false impression of total resection due to lack of visualization of hidden remnants of pathologic tissue, especially in larger cysts, resulting in recurrences. The optimal visualization of surgical field allowed by the EEA may mitigate this under – visualization and, despite cyst capsule resection is often or always not carried out to preserve neurological function, a satisfactory resection with a low risk of recurrence is performed [32]. Imaging surveillance is nonetheless warranted at follow–up.

Advantages of EEA are represented by the short mean surgical time of 111.3 min and LOS of 5.2 days. These results were hampered by the long duration of the procedure #1, which was performed at the beginning of our learning curve in the endoscopic endonasal approach the MC. Gaining further experience with the approach, we observed a marked and progressive reduction of the length of surgery in the following cases (Table 1). A short length of stay (LOS), facilitated by a similarly brief surgical time, is pivotal in minimizing hospital-related complications and healthcare costs, while also improving patient satisfaction—core objectives in the era of Enhanced Recovery After Surgery (ERAS) [38, 39]. Conversely, a transcranial approach to the paramedian skull base, which necessitates violation of the calvarium and epicranial tissues, as well as brain retraction and neurovascular manipulation, is unlikely to meet these goals [13, 40].

In our series, an improvement of the perioperative trigeminal neuralgia was reported in all procedures and of hypoesthesia and paresthesia in 2 patients. The only patient which experienced a stable trigeminal hypoesthesia has been operated four times, and it is supposable that long – term compression, cyst recurrence multiple surgical procedures and dissection maneuvers in a scarred field could have hampered the nerve function. The improvement rate of neuralgia can be explained by the ECs compressive growth pattern in the Gasserian Ganglion. It is therefore plausible that a decompression, even by the means of a STR, could achieve a symptomatologic relief [1]. We observed new post – operative deficits in three procedures, with permanent or temporary injury to III or VI CNs. As previously stated, in procedure #2 and #4 tenacious adherences were encountered, preventing an efficient and smooth dissection of neoplasm and neurovascular structures. This had, in our opinion, a crucial role in the causation of the observed postoperative III CN palsy. These deficits were however transient, probably caused by manipulation rather than by complete injury, in the majority (66.7%) of procedures. Utmost care must be paid during the curetting maneuvers in the antero – superior aspect of the EC, in the V1 region, to avoid injuries of III and VI CN at the superior orbital fissure [28].

The overall incidence of complications was very low, both in our series and in literature. We observed a case of CSF leak in a patient undergoing a second procedure for a recurrence, where the unavoidable scarring tissue and the anatomical disruption could have prevented a correct engraftment of the naso-septal flap.

Preoperative careful surgical planning and meticulous knowledge of the skull base anatomy are mandatory to safely perform an EEA directed to the MC. Its accessing “front – door”, the quadrangular space, is, in normal anatomic conditions, almost virtual and delimitated by delicate neurovascular structure causing destructive consequences if injured. ECs of MC significantly enlarge it, displacing peripherally these structures and especially the ICA in a medial direction in all cases, as showed in Fig. 3, opening therefore the “ventral door” [24, 28, 33, 41, 42]. Neuronavigation with CT angiogram and intraoperative doppler probe are irreplaceable intraoperative adjunct tools which must be implemented in every procedure to minimize neurovascular injuries and postoperative deficits.

According to our results and experience, EEA seems to have peculiar features and advantages favoring its choice in the treatment of ECs of MC, when deemed feasible. The ICA medialization, related to the frequently – observed centrifugal growth pattern of ECs, as in our series, represents the key anatomical enabler of a viable endonasal corridor. Moreover, the keratinaceous content of ECs is usually soft and can be for the largest part easily removed with suction from an endoscopic endonasal route. With the development of advanced skull base reconstruction techniques, the risk of CSF leak can be significantly decreased, and, when it occurs, prompt surgical revision prevents the development of meningitis (which was never observed in our series). Its main and unavoidable limitation is its poor visualization, exposure and surgical maneuverability when addressing lesions with extension in the posterior fossa and CPA, as observable in some ECs [18, 31, 43–45], or potentially with lateral extension. If the ICA is not medialized by the neoplasm’s growth pattern and the “quadrangular space” is narrow, the endoscopic endonasal approach is not feasible. In those patients with significant lateral extension, or with a lateral ICA preventing EEA, a transcranial microsurgical route, via a pterional approach, can be considered on a careful anatomy - based case – by – case selection [13, 46]. Transcranial approaches are unavoidably burdened by the brain retraction associated morbidity, which is prevented by EEA, and the surgical cave can be under – visualized when compared to the endoscopic view, increasing the risk of an incomplete resection, as previously discussed [13, 32]. On the other hand, MC ECs with significant CPA extension could be in our opinion approached with combined multistep approaches. An option is the resection of the MC portion of the cyst followed by a wait – and – see strategy with yearly MRI imaging, feasible especially in those cases when the remnant is small and possible preoperative debilitating symptomatology, such as trigeminal neuralgia, has regressed. Conversely, an upfront second step surgical resection of the CPA remnant, through a lateral skull base or a retrosigmoid approach, should be performed when the remnant is large, possibly abutting the brainstem, and the symptomatology has not regressed.

During the last decade, the endoscopic transorbital approach (ETOA) was developed and has been gaining clinical validation and popularity in the surgical treatment of Meckel’s cave neoplasms, mainly schwannomas. It provides a lateral-to-medial minimally invasive route through a superior eyelid incision, with access to middle fossa achieved with drilling of the greater sphenoidal wing, dissection of the meningo-orbital band and exposure of the lateral wall of the cavernous sinus and the lateral compartment of Meckel’s cave [14, 47–54]. Various anatomical studies investigated also variants and extension of the endoscopic approach, in order to improve the surgical maneuverability and exposure and to assess its posterior extension to petrous apex and posterior fossa [55–60]. To our knowledge, no studies have reported yet the resection of a MC EC via an ETOA, and hypothesis can be made from the evidence reported for the treatment of other neoplasms [51, 53, 56, 58, 61]. In our opinion, from a surgical standpoint, given the soft consistence of the cyst content, this could be a valid alternative to EEA, harboring an unavoidable risk of ocular and orbital morbidity, such as ptosis and diplopia, but conversely minimizing the risk of CSF leak which is intrinsic in every endonasal route [53, 58, 61]. ETOA can be considered, on a case – by – case basis according to surgeon’s confidence and anatomical features of the single patients, when addressing MC neoplasms, especially, in those cases where the medial displacement of the ICA and the latero - superior displacement of trigeminal branches are not enough to “open” an adequate quadrangular space [61]. 

In our analysis, as considered two different pathological entities, we purposely excluded dermoid cysts. They are even rarer lesions (four to nine times less common than epidermoid cysts) and mainly located in the midline. Their occurrence in Meckel’s cave is exceedingly uncommon, with less than anecdotal frequency [32, 62].

The main strength of this study is that all patients were homogenously treated in a referral center for skull base pathologies, with special expertise in endoscopic surgery, by a highly – specialized team of neurosurgeons, ENT surgeons, neuropathologists and neuroradiologists. Despite its retrospective observational design, no patients were excluded from the study or lost at follow–up. Conversely, the rarity of disease precludes us a more detailed evaluation, including statistical analysis to individuate predictors of outcome and complication. Similarly, the scarcity and the heterogeneity of the available literature, composed by case reports and lacking in many cases solid follow–up data, precludes an effective overall analysis. Moreover, when available, substantial heterogeneity was observed in the reporting of the lesion size, such as the volume, two dimensions or the largest diameter.

## Conclusions

ECs primarily arising from MC are rare lesions, with only few cases reported in the current literature. EEA is valid approach for their treatment, allowing an effective and safe resection. Despite their indolent clinical behavior, GTR should be pursued whenever feasible, without injuring neurovascular structures, as surgical resection of recurrences could be burdened by a higher risk of complications and reduced extent of removal for the presence of scar or fibrous tissue. The reduced invasiveness of EEA allows a fast recovery of the patient with a short hospital LOS. Further studies, with larger caseloads and long – term follow up, are warranted to confirm these results, also directly comparing outcomes and indications of EEA with other approaches such as ETOA.

## Supplementary Information

Below is the link to the electronic supplementary material.

Supplementary Material 1

## Data Availability

No datasets were generated or analysed during the current study.

## References

[1] Busch CM, Prickett JT, Stein R, Cuoco JA, Marvin EA, Witcher MR (2019) Meckel cave epidermoid cyst presenting as multiple cranial nerve deficits due to indirect tumoral compression of the cavernous sinus: A case report and literature review. World Neurosurg 121:88–94. 10.1016/j.wneu.2018.09.206 30308341 10.1016/j.wneu.2018.09.206

[2] Mehta DS, Malik GB, Dar J (1971) Trigeminal neuralgia due to cholesteatoma of meckel’s cave. Case report. J Neurosurg 34:572–574. 10.3171/jns.1971.34.4.0572 5554365 10.3171/jns.1971.34.4.0572

[3] Arai A, Sasayama T, Koyama J, Fujita A, Hosoda K, Kohmura E (2010) Epidermoid cyst in meckel’s cave with unusual computed tomography and magnetic resonance imaging findings. Case report. Neurol Med Chir (Tokyo) 50:701–704. 10.2176/nmc.50.701 20805660 10.2176/nmc.50.701

[4] Beck DW, Menezes AH (1987) Lesions in meckel’s cave: variable presentation and pathology. J Neurosurg 67:684–689. 10.3171/jns.1987.67.5.0684 3668636 10.3171/jns.1987.67.5.0684

[5] Furtado SV, Hegde AS (2009) Trigeminal neuralgia due to a small meckel’s cave epidermoid tumor: surgery using an extradural corridor. Skull Base 19:353–357. 10.1055/s-0029-1220201 20190946 10.1055/s-0029-1220201 PMC2765701

[6] Gohil J, Rajasekar G, Shivhare P, Nair P, Abraham M (2019) A rare case of an extensive Multi-compartment epidermoid presenting with pure motor trigeminal Neuropathy, case report and review of literature. J Neurosci Rural Pract 10:364–366. 10.4103/jnrp.jnrp_277_18 31001038 10.4103/jnrp.jnrp_277_18 PMC6454975

[7] Kapila A, Steinbaum S, Chakeres DW (1984) Meckel’s cave epidermoid with trigeminal neuralgia: CT findings. J Comput Assist Tomogr 8:1172–1174. 10.1097/00004728-198412000-00027 6501628 10.1097/00004728-198412000-00027

[8] Lopez Gomez P, Mato Mañas D, Marco de Lucas E (2021) Epidermoid cyst extending along trigeminal nerve pathway with unusual imaging findings. World Neurosurg 146:75–77. 10.1016/j.wneu.2020.10.148 33144211 10.1016/j.wneu.2020.10.148

[9] Morisako H, Sasaki T, Ikegami M, Tanoue Y, Ohata H, Goudihalli SR, Fernandez-Miranda JC, Ohata K, Goto T (2024) Purely endoscopic subtemporal keyhole anterior transpetrosal approach to access the petrous apex region: surgical techniques and early results. J Neurosurg 141:752–761. 10.3171/2024.1.JNS231774 38579340 10.3171/2024.1.JNS231774

[10] Nadkarni T, Dindorkar K, Muzumdar D, Goel A (2000) Epidermoid tumor within meckel’s cave–case report. Neurol Med Chir (Tokyo) 40:74–76. 10.2176/nmc.40.74 10721260 10.2176/nmc.40.74

[11] Sharifi G, Mohammadi E, Jafari A, Jamali E, Sabouri S, Akbari N (2022) A case of meckel’s cave epidermoid cyst with unilateral abducens nerve paresis as the sole presentation. Interdisciplinary Neurosurg 30:101613. 10.1016/j.inat.2022.101613

[12] Sun DQ, Menezes AH, Howard MA, Gantz BJ, Hasan DM, Hansen MR (2018) Surgical management of tumors involving meckel’s cave and cavernous sinus: role of an extended middle fossa and lateral sphenoidectomy approach. Otol Neurotol 39:82–91. 10.1097/MAO.0000000000001602 29135804 10.1097/MAO.0000000000001602 PMC6042969

[13] Van Rompaey J, Bush C, Khabbaz E, Vender J, Panizza B, Solares CA (2013) What is the best route to the Meckel cave? Anatomical comparison between the endoscopic endonasal approach and a lateral approach. J Neurol Surg B Skull Base 74:331–336. 10.1055/s-0033-1342989 24436933 10.1055/s-0033-1342989 PMC3836809

[14] Zanin L, Agosti E, Ebner F, de Maria L, Belotti F, Buffoli B, Rezzani R, Hirt B, Ravanelli M, Ius T, Zeppieri M, Tatagiba MS, Fontanella MM, Doglietto F (2023) Quantitative anatomical comparison of surgical approaches to meckel’s cave. J Clin Med 12:6847. 10.3390/jcm12216847 37959312 10.3390/jcm12216847 PMC10648058

[15] Kalani MYS, Couldwell WT (2018) Retrosigmoid craniotomy for resection of an epidermoid cyst of the posterior fossa. J Neurol Surg B Skull Base 79:S411–S412. 10.1055/s-0038-1669980 30456045 10.1055/s-0038-1669980 PMC6240416

[16] KATSUKI M, NARITA N, YASUDA I, TOMINAGA T (2021) A case of trigeminal neuralgia due to cerebellopontine epidermoid cyst: discrepancy between intraoperative and radiological findings of constructive interference in steady state (CISS). NMC Case Rep J 8:551–556. 10.2176/nmccrj.cr.2021-0035 35079516 10.2176/nmccrj.cr.2021-0035 PMC8769480

[17] Sutiono AB, Sidabutar R, Pareira ES, Toda M, Yoshida K (2019) Characteristics intracranial epidermoid cyst between two hospital from developed vs developing institution and literature review. Interdisciplinary Neurosurg 18:100500. 10.1016/j.inat.2019.100500

[18] Matsushima K, Kohno M, Nakajima N, Ichimasu N (2019) Combined transpetrosal approach with preservation of superior petrosal vein drainage for a cerebellopontine angle epidermoid cyst extending into meckel’s cave: 3-Dimensional operative video. Oper Neurosurg (Hagerstown) 16:E172–E173. 10.1093/ons/opy286 10.1093/ons/opy286 30452703

[19] Samii M, Tatagiba M, Piquer J, Carvalho GA (1996) Surgical treatment of epidermoid cysts of the cerebellopontine angle. J Neurosurg 84:14–19. 10.3171/jns.1996.84.1.0014 8613823 10.3171/jns.1996.84.1.0014

[20] Tatagiba M, Iaconetta G, Samii M (2000) Epidermoid cyst of the cavernous sinus: clinical features, pathogenesis and treatment. Br J Neurosurg 14:571–575. 10.1080/02688690050206747 11272040 10.1080/02688690050206747

[21] Cárdenas Ruiz-Valdepeñas E, Simal Julián JA, Pérez Prat G, Arraez MA, Ambrosiani J, Martin Schrader I, Soto Moreno A, Kaen A (2022) The quadrangular Space, endonasal access to the Meckel cave: technical considerations and clinical series. World Neurosurg 163:e124–e136. 10.1016/j.wneu.2022.03.077 35331950 10.1016/j.wneu.2022.03.077

[22] de Lara D, Ditzel Filho LFS, Prevedello DM, Carrau RL, Kasemsiri P, Otto BA, Kassam AB (2014) Endonasal endoscopic approaches to the paramedian skull base. World Neurosurg 82:S121–129. 10.1016/j.wneu.2014.07.036 25496623 10.1016/j.wneu.2014.07.036

[23] Ferlendis L, Bossi B, Barillot C, Leocata A, Veiceschi P, Pozzi F, Castelnuovo P, Locatelli D (2024) Endoscopic transpterygoid approach to meckel’s cave: technical considerations and retrospective analysis of a clinical series. Clin Neurol Neurosurg 243:108382. 10.1016/j.clineuro.2024.108382 38944020 10.1016/j.clineuro.2024.108382

[24] Jouanneau E, Simon E, Jacquesson T, Sindou M, Tringali S, Messerer M, Berhouma M (2014) The endoscopic endonasal approach to the meckel’s cave tumors: surgical technique and indications. World Neurosurg 82:S155–161. 10.1016/j.wneu.2014.08.003 25107326 10.1016/j.wneu.2014.08.003

[25] Kassam AB, Prevedello DM, Carrau RL, Snyderman CH, Gardner P, Osawa S, Seker A, Rhoton AL (2009) The front door to meckel’s cave: an anteromedial corridor via expanded endoscopic endonasal approach- technical considerations and clinical series. Neurosurgery 64:ons71-82; discussion ons82-83. 10.1227/01.NEU.0000335162.36862.54 10.1227/01.NEU.0000335162.36862.54 19240575

[26] Raza SM, Donaldson AM, Mehta A, Tsiouris AJ, Anand VK, Schwartz TH (2014) Surgical management of trigeminal schwannomas: defining the role for endoscopic endonasal approaches. Neurosurg Focus 37:E17. 10.3171/2014.7.FOCUS14341 25270136 10.3171/2014.7.FOCUS14341

[27] Yang L, Hu L, Zhao W, Zhang H, Liu Q, Wang D (2018) Endoscopic endonasal approach for trigeminal schwannomas: our experience of 39 patients in 10 years. Eur Arch Otorhinolaryngol 275:735–741. 10.1007/s00405-018-4871-1 29350272 10.1007/s00405-018-4871-1

[28] Zoli M, Ratti S, Guaraldi F, Milanese L, Pasquini E, Frank G, Billi AM, Manzoli L, Cocco L, Mazzatenta D (2018) Endoscopic endonasal approach to primitive meckel’s cave tumors: a clinical series. Acta Neurochir (Wien) 160:2349–2361. 10.1007/s00701-018-3708-4 30382359 10.1007/s00701-018-3708-4

[29] Aldea S, Veyrat M, Bourdillon P, Ayache D, Le Guérinel C (2022) Extended endoscopic endonasal approach for a giant parasellar epidermoid cyst. J Neurol Surg B Skull Base 83:e653–e654. 10.1055/s-0041-1727127 36068911 10.1055/s-0041-1727127 PMC9440980

[30] Morshed RA, El-Sayed IH, Goldschmidt E (2022) Endoscopic endonasal transpterygoid approach for resection of a Meckel cave epidermoid cyst: 2-Dimensional operative video. Oper Neurosurg (Hagerstown) 23:e122. 10.1227/ons.0000000000000234 10.1227/ons.0000000000000234 35838467

[31] Zakaria J, Saini P, Yanovskaya M, Tsiang JT, Ravindran K, Johans S, Patel CR, Germanwala AV (2020) Endoscopic endonasal resection of meckel’s cave epidermoid cysts: case discussion and literature review. Case Rep Neurol Med 2020:7853279. 10.1155/2020/7853279 32089913 10.1155/2020/7853279 PMC7029282

[32] Vaz-Guimaraes F, Koutourousiou M, de Almeida JR, Tyler-Kabara EC, Fernandez-Miranda JC, Wang EW, Snyderman CH, Gardner PA (2019) Endoscopic endonasal surgery for epidermoid and dermoid cysts: a 10-year experience. J Neurosurg 130:368–378. 10.3171/2017.7.JNS162783 29547084 10.3171/2017.7.JNS162783

[33] Dolci RLL, Ditzel Filho LFS, Goulart CR, Upadhyay S, Buohliqah L, Lazarini PR, Prevedello DM, Carrau RL (2018) Anatomical nuances of the internal carotid artery in relation to the quadrangular space. J Neurosurg 128:174–181. 10.3171/2016.10.JNS16381 28298027 10.3171/2016.10.JNS16381

[34] Zhou F, Yang Z, Zhu W, Chen L, Song J, Quan K, Li S, Li P, Pan Z, Liu P, Mao Y (2018) Epidermoid cysts of the cavernous sinus: clinical features, surgical outcomes, and literature review. J Neurosurg 129:973–983. 10.3171/2017.6.JNS163254 29271707 10.3171/2017.6.JNS163254

[35] Iaconetta G, Carvalho GA, Vorkapic P, Samii M (2001) Intracerebral epidermoid tumor: a case report and review of the literature. Surg Neurol 55:218–222. 10.1016/s0090-3019(01)00346-9 11358593 10.1016/s0090-3019(01)00346-9

[36] Lopes M, Capelle L, Duffau H, Kujas M, Sichez J-P, Van Effenterre R, Faillot T, Bitar A, Fohanno D (2002) [Surgery of intracranial epidermoid cysts. Report of 44 patients and review of the literature]. Neurochirurgie 48:5–13 11972145

[37] Schiefer TK, Link MJ (2008) Epidermoids of the cerebellopontine angle: a 20-year experience. Surg Neurol 70:584–590 discussion 590. 10.1016/j.surneu.2007.12.021 18423548 10.1016/j.surneu.2007.12.021

[38] Cossu G, Belouaer A, Kloeckner J, Caliman C, Agri F, Daniel RT, Gaudet JG, Papadakis GE, Messerer M (2023) The enhanced recovery after surgery protocol for the perioperative management of pituitary neuroendocrine tumors/pituitary adenomas. Neurosurg Focus 55:E9. 10.3171/2023.9.FOCUS23529 38039521 10.3171/2023.9.FOCUS23529

[39] Shah H, Slavin A, Botvinov J, O’Malley GR, Sarwar S, Patel NV (2024) Endoscopic endonasal transsphenoidal surgery for the resection of pituitary adenomas: A prime candidate for a shortened length of stay enhanced recovery after surgery protocol? A systematic review. World Neurosurg 186:145–154. 10.1016/j.wneu.2024.03.135 38552787 10.1016/j.wneu.2024.03.135

[40] Dasenbrock HH, Liu KX, Devine CA, Chavakula V, Smith TR, Gormley WB, Dunn IF (2015) Length of hospital stay after craniotomy for tumor: a National surgical quality improvement program analysis. Neurosurg Focus 39:E12. 10.3171/2015.10.FOCUS15386 26621410 10.3171/2015.10.FOCUS15386

[41] Cebula H, Kurbanov A, Zimmer LA, Poczos P, Leach JL, De Battista JC, Froelich S, Theodosopoulos PV, Keller JT (2014) Endoscopic, endonasal variability in the anatomy of the internal carotid artery. World Neurosurg 82:e759–764. 10.1016/j.wneu.2014.09.021 25238676 10.1016/j.wneu.2014.09.021

[42] Dolci RLL, Upadhyay S, Ditzel Filho LFS, Fiore ME, Buohliqah L, Lazarini PR, Prevedello DM, Carrau RL (2016) Endoscopic endonasal study of the cavernous sinus and quadrangular space: anatomic relationships. Head Neck 38(Suppl 1):E1680–1687. 10.1002/hed.24301 26875705 10.1002/hed.24301

[43] Das P, Borghei-Razavi H, Moore NZ, Recinos PF (2019) Posterior approach to meckel’s cave: retrosigmoid craniectomy with endoscopic assistance. J Neurol Surg B Skull Base 80:S331–S332. 10.1055/s-0039-1677851 31143619 10.1055/s-0039-1677851 PMC6534686

[44] Hitti FL, Lee JYK (2019) Endoscopic resection of a cerebellopontine angle epidermoid cyst via a retrosigmoid approach. J Neurol Surg B Skull Base 80:S330. 10.1055/s-0039-1677852 31143618 10.1055/s-0039-1677852 PMC6534687

[45] Vakharia KV, Naylor RM, Nassiri AM, Driscoll CLW, Link MJ (2021) Microsurgical resection of a petroclival epidermoid cyst using an anterior petrosectomy approach: 2-Dimensional operative video. Oper Neurosurg (Hagerstown) 21:E565. 10.1093/ons/opab364 10.1093/ons/opab364 34560780

[46] Roche P-H, Troude L, Peyriere H, Noudel R (2014) The epidural approach to the meckel’s cave: a how I do it. Acta Neurochir 156:217–220. 10.1007/s00701-013-1916-5 24193888 10.1007/s00701-013-1916-5

[47] Corrivetti F, de Notaris M, Seneca V, Di Nuzzo G, Catapano G (2024) Is it time for a paradigm shift in the surgical management of trigeminal schwannomas? Evaluating the role of the transorbital approach: an anatomo-Clinical study and systematic literature review. World Neurosurg 190:e1025–e1037. 10.1016/j.wneu.2024.08.055 39151698 10.1016/j.wneu.2024.08.055

[48] Corvino S, Kassam A, Piazza A, Corrivetti F, Spiriev T, Colamaria A, Cirrottola G, Cavaliere C, Esposito F, Cavallo LM, Iaconetta G, de Notaris M (2024) Open-door extended endoscopic transorbital technique to the paramedian anterior and middle cranial fossae: technical notes, anatomomorphometric quantitative analysis, and illustrative case. Neurosurg Focus 56:E7. 10.3171/2024.1.FOCUS23838 38560942 10.3171/2024.1.FOCUS23838

[49] Di Somma A, Langdon C, de Notaris M, Reyes L, Ortiz-Perez S, Alobid I, Enseñat J (2021) Combined and simultaneous endoscopic endonasal and transorbital surgery for a meckel’s cave schwannoma: technical nuances of a mini-invasive, multiportal approach. J Neurosurg 134:1836–1845. 10.3171/2020.4.JNS20707 32650309 10.3171/2020.4.JNS20707

[50] Han X, Yang H, Wang Z, Li L, Li C, Han S, Wu A (2023) Endoscopic transorbital approach for skull base lesions: a report of 16 clinical cases. Neurosurg Rev 46:74. 10.1007/s10143-023-01980-y 36947242 10.1007/s10143-023-01980-y

[51] Jeon C, Hong C-K, Woo KI, Hong SD, Nam D-H, Lee J-I, Choi JW, Seol HJ, Kong D-S (2019) Endoscopic transorbital surgery for meckel’s cave and middle cranial fossa tumors: surgical technique and early results. J Neurosurg 131:1126–1135. 10.3171/2018.6.JNS181099 30544350 10.3171/2018.6.JNS181099

[52] Kong D-S, Shin HJ (2024) Endoscopic transorbital surgery for trigeminal schwannoma: introduction of a novel approach: 2-Dimensional operative video. Oper Neurosurg (Hagerstown) 26:96–97. 10.1227/ons.0000000000000893 10.1227/ons.0000000000000893 37819070

[53] Mathios D, Bobeff EJ, Longo D, Nilchian P, Estin J, Schwartz AC, Austria Q, Anand VK, Godfrey KJ, Schwartz TH (2024) The lateral transorbital approach to the medial sphenoid wing, anterior clinoid, middle fossa, cavernous sinus, and meckel’s cave: target-based classification, approach-related complications, and intermediate-term ocular outcomes. J Neurosurg 140:677–687. 10.3171/2023.6.JNS23678 37657097 10.3171/2023.6.JNS23678

[54] Salem EH, Abd El-Fattah AM, Ebada HA, van Koevering K, Hardesty DA, Prevedello DM, Al-Saddeik MAE-H, Carrau RL (2024) Endoscopic multiportal approaches to meckel’s cave: A cadaveric study and a Three-Dimensional anatomical video. J Neurol Surg B Skull Base 85:641–649. 10.1055/a-2158-6037 39483170 10.1055/a-2158-6037 PMC11524734

[55] Carretta A, Magnani M, Sollini G, Pasquini E, Rustici A, Neri I, Manzoli L, Ratti S, Mazzatenta D, Zoli M (2024) Advantages and limitations of orbital rim resection in transorbital endoscopic approach: an anatomical study. Acta Neurochir (Wien) 166:501. 10.1007/s00701-024-06397-0 39672963 10.1007/s00701-024-06397-0

[56] De Simone M, Zoia C, Choucha A, Kong D-S, De Maria L (2024) The transorbital approach: A comprehensive review of Targets, surgical Techniques, and multiportal variants. J Clin Med 13:2712. 10.3390/jcm13092712 38731240 10.3390/jcm13092712 PMC11084817

[57] Hong C-K, Mosteiro A, Kong D-S, Tafuto R, Codes M, Ferres A, Matas J, Manfrellotti R, Prats-Galino A, Di Somma A, Enseñat J (2024) Endoscopic transorbital approach to the petrous apex: is orbital rim removal worthwhile for the exposure? An anatomical study with illustrative case. J Neurosurg 1–9. 10.3171/2024.3.JNS232834 10.3171/2024.3.JNS232834 38875727

[58] Kong D-S, Kim YH, Lee W-J, Kim Y-H, Hong C-K (2023) Indications and outcomes of endoscopic transorbital surgery for trigeminal Schwannoma based on tumor classification: a multicenter study with 50 cases. J Neurosurg 138:1653–1661. 10.3171/2022.9.JNS22779 36681991 10.3171/2022.9.JNS22779

[59] Kong D-S, Lee WJ, Kim GJ, Hong C-K (2024) The feasibility and clinical outcome of endoscopic transorbital transcavernous approaches with or without petrosectomy for petroclival lesions. J Neurosurg 1–8. 10.3171/2024.6.JNS232976 10.3171/2024.6.JNS232976 39504547

[60] Zoia C, Mastantuoni C, Solari D, de Notaris M, Corrivetti F, Spena G, Cavallo LM (2024) Transorbital and supraorbital uniportal multicorridor approach to the orbit, anterior, middle and posterior cranial fossa: anatomic study. Brain Spine 4:102719. 10.1016/j.bas.2023.102719 38163002 10.1016/j.bas.2023.102719 PMC10753433

[61] Park HH, Hong SD, Kim YH, Hong C-K, Woo KI, Yun I-S, Kong D-S (2020) Endoscopic transorbital and endonasal approach for trigeminal schwannomas: a retrospective multicenter analysis (KOSEN-005). J Neurosurg 133:467–476. 10.3171/2019.3.JNS19492 31226689 10.3171/2019.3.JNS19492

[62] Patel BK, Darshan HR, Binu A, George T, Easwer HV, Nair P (2022) Endoscopic endonasal excision of a meckel’s cave dermoid cyst. Neurol India 70:884–889. 10.4103/0028-3886.349623 35864614 10.4103/0028-3886.349623

